# Complete blood count alterations in COVID-19 patients: A narrative review

**DOI:** 10.11613/BM.2021.030501

**Published:** 2021-10-15

**Authors:** Mariangela Palladino

**Affiliations:** Emergency Department, Umberto I University Hospital, Rome, Italy

**Keywords:** haematology, COVID-19 virus disease, critical care

## Abstract

Coronavirus disease 2019 (COVID-19) pandemic represents a scientific and social crisis. One of the main unmet needs for coronavirus disease 2019 is its unpredictable clinical course, which can rapidly change in an irreversible outcome. COVID-19 patients can be classified into mild, moderate, and severe. Several haematological parameters, such as platelets, white blood cell total count, lymphocytes, neutrophils, (together with neutrophil-lymphocyte and platelet-lymphocyte ratio), and haemoglobin were described to be associated with COVID-19 infection and severity. The purpose of these review is to describe the current state of the art about complete blood count alterations during COVID-19 infection, and to summarize the crucial role of some haematological parameters during the course of the disease. Decreased platelet, lymphocyte, haemoglobin, eosinophil, and basophil count, increased neutrophil count and neutrophil-lymphocyte and platelet-lymphocyte ratio have been associated with COVID-19 infection and a worse clinical outcome. Our study adds some novelty about the identification of effective biomarkers of progressive disease, and might be helpful for diagnosis, prevention of complications, and effective therapy.

## Introduction

In December 2019, coronavirus disease 2019 (COVID-19) was firstly reported in Wuhan, China. The pathogenic agent was determined to be a novel b-coronavirus, which is currently named severe acute respiratory syndrome coronavirus 2 (SARS-CoV-2) (*1*). Patients experience a spectrum of disease from a mild illness, through varying severity of pneumonia all the way to acute respiratory distress syndrome (ARDS) and sepsis with multi-organ failure and death. The majority of patients develop pneumonia, which can proceed in up to 20-30% of cases to respiratory failure requiring intubation and ventilatory support (*2*). Because of the rapid spread of the COVID-19 pandemic, affected countries have taken a heterogeneous and evolving approach to the diagnosis of infection in patients, and continue to have different and, in some cases, evolving strategies to determine what segments of the population should be tested. Currently, the gold standard for the diagnosis of COVID‐19 patients is the detection of SARS‐CoV‐2 nucleic acid by real‐time reverse transcription polymerase chain reaction (RT-PCR) in respiratory specimens (oro- and nasopharyngeal swabs, sputa, nasal aspirates and washes, bronchoalveolar lavage and lung tissue specimens collected at autopsy) (*3*). Based on studies conducted in China and elsewhere, the clinical haematology laboratory plays an important role by providing the clinical team a number of useful prognostic markers and might be an important partner in the triage and management of affected patients. Some laboratory abnormalities include the decreased white blood cell and lymphocyte count, neutrophilia, thrombocytopenia, increased C‐reactive protein (CRP), erythrocyte sedimentation rate (ESR), and abnormal procalcitonin (PCT) in most patients (*4*). In particular, the blood count of patients with COVID-19 infection at diagnosis shows alterations that correlate with the stage and severity of the disease (*5*).

Laboratory blood tests have not been assessed with regard to their sensitivity or specificity for the diagnosis of COVID-19, nor their value as prognostic indicators. This literature review summarizes the blood cell count variations and their clinical significance in COVID-19 patients, having the purpose to gain an understanding of the existing data about haematological alterations occurring in COVID-19 infection, and debating particular prognostic markers which may be useful to stratify patients at the early diagnosis and eventually provide prompt treatment.

## Literature search

A literature search was performed using the PubMed and Google Scholar databases to identify studies on haematological findings of COVID-19 up to April 2021. The keywords used in the search were as follows: SARS-CoV-2, COVID-19, blood count, laboratory findings, and haematology. The initial selection was based on the article title and abstract, following which the full-text article was read. Inclusion criteria were any study or article that included information about laboratory alterations in SARS-CoV-2. All types of studies and countries of origin were eligible for inclusion. We excluded articles that exclusively reported data on coagulative parameters, or that did not relate to the clinical aspects of interest, and that were not written in the English language. The findings from primary research articles, reviews, case reports, and case series were summarized and discussed narratively. Our search retrieved 446 records, of which 253 were excluded *via* title and abstract screening. A total of 193 full articles were assessed for eligibility. Of these, 120 papers relating to blood cells and SARS-CoV-2 were identified, from which data were extracted and synthesized. Articles relevant to the topic were included in the reference lists, after removing duplicates.

## Platelets

Platelet (PLT) count is an important parameter included in numerous classification systems that evaluate disease severity, such as in multiorgan dysfunction syndrome. In COVID-19 infection, the presence of thrombocytopenia correlates with the severity of the disease and indicates the presence of a consumption coagulopathy. Similar to published data on severe acute respiratory syndrome and Middle East respiratory syndrome infection, it is found in approximately 60% of severe patients (*6*). Lippi *et al.* correlated a low PLT count with higher mortality and more severe COVID-19 illness (*7*). Platelet number was found to be lower in patients with either more severe illness or poor outcomes and even lower in non-survivors. An interesting point of the subgroup analysis is that thrombocytopenia may not be significantly related to intensive care unit (ICU) admission. This aspect could be explained considering that thrombocytopenia tends to reach a significant level in the late clinical stage of COVID-19 (*8*).

The mechanism by which the coronavirus interferes with the haematopoietic system is still unclear. Three mechanisms of a cascade can be assumed to explain thrombocytopenia in SARS-CoV-2 infections: 1) direct infection of bone marrow cells by the virus with inhibition of PLT synthesis; 2) destruction of PLTs by the immune system; 3) aggregation of PLTs in the lungs with the formation of microthrombi and further consumption of PLTs.

Viruses can interact with megakaryocytes and reduce PLT synthesis (*9*). It has been assumed that the SARS-CoV-2 inhibits bone marrow haematopoiesis through specific receptors to depress the primary formation of PLTs and resulting thrombocytopenia (*10*). Viral infection and inflammation result in pulmonary capillary damage. Damaged lung tissues and pulmonary endothelial cells may cause a process of megakaryocyte rupture and increased PLT consumption (*11*). Also, SARS-CoV-2 can boost autoantibodies and immune complexes, resulting in the specific destruction of PLTs by the immune system (*12*). While PLTs contribute to the basal barrier integrity of the alveolar capillaries, they may also contribute to lung injury in a variety of pulmonary disorders and syndromes (*13*). PLT-leukocyte aggregates and PLT-endothelial interactions appear to play a role in the pathogenesis of acute lung injury (*14*). In Covid-19 infection, the damage of the lung tissues and lung endothelial cells can cause PLT aggregations with the formation of micro thrombi and further consumption of PLTs (*15*). In fact, most patients with thrombocytopenia have high concentrations of D-dimers with alteration of the coagulation parameters that confirm the hypothesis of triggering intravascular coagulation (*16*).

The PLT count associated with the hypoxemia value has also been described as a predictive model of the severity of the disease, with an accuracy of 96% (*17*). Conversely, only a small fraction of patients have thrombocytosis (*18*). COVID-19 patients were found to have significantly larger mean platelet volume (MPV) than critically ill non-COVID-19 patients matched for PLT count (11.6 *vs* 10.5 fL, P = 0.013). Moreover, there was a significant trend of elevated immature platelet fraction (IPF) in COVID-19 patients. COVID-19 positivity was highly predictive of an absolute IPF of 7.5 x10^9^/L or higher; also, COVID-19-positive patients had relative IPF ≥ 8% at PLT counts up to 251 x10^9^/L. In contrast, in the non-COVID-19 patients, a relative IPF of ≥ 8% was restricted to those with PLT counts less than 70 x10^9^/L (*19*). These findings suggest that COVID-19 is associated with the increased production of large immature PLTs, as megakaryocytes respond to increased PLT consumption.

## White blood cells

### Lymphocytes

The best recognized haematological abnormality in patients affected by COVID-19 infection is lymphopenia, which is seen in up to 85% of severe cases with the severity of lymphopenia linked to outcome (*20*). The presence of lymphopenia (defined by an absolute number of lymphocytes < 1.0 x10^9^/L) is reported in most of the published series and is commonly considered to be a deficient immunological response to viral infection (*21*).

Since the SARS-CoV-2 was identified, this novel virus began to be compared to influenza. Of respiratory infections, influenza is the most well studied viral infection and is commonly reported as the cause of epidemics. The lymphocyte count of the severe COVID-19 group was found to be significantly lower than in the common COVID-19 and influenza groups (P < 0.001 and P = 0.012). Common cases of Covid-19 infection were defined as those who had a fever, respiratory tract symptoms, and pneumonia on imaging. Severe cases were those who had one of the following three clinical manifestations: (a) shortness of breath with a respiratory rate greater than 30 breaths/min, (b) mean oxygen saturation ≤ 93% in the resting state, and (c) partial pressure of arterial oxygen/oxygen concentration ≤ 300 mmHg. Severe cases also included the progress of lesions by more than 50% within 24 to 48 hours, as detected by pulmonary imaging. All influenza cases were confirmed by RT‐PCR assay of nasal and pharyngeal swab specimens. Lymphocytes percentage rate of influenza and severe COVID‐19 groups were significantly lower than in the common COVID‐19 group (P < 0.001) (*22*). In a cohort of 120 COVID-19 patients, 100 influenza patients, and 61 healthy controls, lower lymphocytes were found in COVID-19 and influenza groups compared to healthy controls (*23*). However, distinct subpopulations of CD8+ T cells might be involved in COVID-19 and influenza. When analysing disease-specific transcriptional signatures in CD8+ T cells, the authors found that biological pathways for responses to interferon (IFN)-I and -II were more associated with the influenza-specific cellular cluster, whereas pathways for the response to tumour necrosis factor (TNF) or interleukin-1β (IL-1β) were more prominent in COVID-19-specific clusters (*24*). In summary, COVID‐19 and influenza can cause different changes in peripheral blood parameters, which should be considered in the early stages of COVID‐19 and influenza.

It is well known that alteration in total lymphocyte numbers and the subsets varies with different virus types, indicating a potential association between lymphocyte subset alteration and viral pathogenic mechanisms (*25*). Lymphopenia might be caused by virus attachment or indirectly by immune injuries from inflammatory mediators. Moreover, exudation of circulating lymphocytes into inflammatory lung tissues might also lead to lymphopenia. The reduction of lymphocyte subset count in COVID-19 patients was investigated across 20 peer-reviewed studies for reporting lymphocyte subset counts and COVID-19 disease severity. CD4+ T cell, CD8+ T cell, B cell, Natural killer (NK) cell, and total lymphocyte cell counts all showed a statistically significant reduction in patients with severe/critical COVID-19 disease compared to mild/moderate disease (*26*). The results remained consistent despite the differences in the definition of disease severity across the studies (*27*-*29*). Counts of total, CD4+, or CD8+ T cells lower than 800, 400, or 300/µL, respectively, were negatively correlated with the patient outcome; the counts of the total, CD4+, and CD8+ T cells, importantly, were significantly lower in ICU patients than non-ICU cases. Further, an age-dependent reduction of T cell numbers was observed, with the lowest T cells numbers found in patients ≥ 60 years old, thus suggesting a potential cause for increased susceptibility in elderly patients (*27*). No significant difference was observed in CD4+/CD8+ ratio and NK cells in severe cases compared with mild illness (*28*), (Table 1). Total lymphocytes, CD4+, and CD8+ T cells were also found to inversely correlate with inflammatory indicators as ESR and CRP, while CD4+/CD8+ ratio was directly correlated with ESR and CRP (*28*). However, the expression of cell surface and intracellular molecules is not detectable by routine automated cell counter but needs to be analysed by flow cytometry.

**Table 1:** Findings for CD4+, CD8+, CD4+/CD8+ ratio, NK cells variations in severe/critical COVID-19 disease compared to mild/moderate disease

**First Author (Country)** **(Reference number)**	**Sample size (N)**	**Total lymphocytes** **(P-value)**	**T cells** **(P-value)**	**CD4+ cells** **(P-value)**	**CD8+ cells** **(P-value)**	**CD4+/CD8+ ratio** **(P-value)**	**NK cells** **(P-value)**	**B cells** **(P-value)**
Huang *et al.* (USA) (26)	3017	decreased (< 0.001)	n.a.	decreased (< 0.001)	decreased (< 0.001)	n.a.	decreased (< 0.001)	decreased (< 0.001)
Diao *et al.* (China) (27)	522	n.a.	decreased (< 0.01)	decreased (< 0.01)	decreased (< 0.01)	n.a.	n.a.	n.a.
Wang *et al.* (China) (28)	60	decreased (< 0.001)	n.a.	decreased (0.024)	decreased (0.005)	no difference (0.392)	no difference (0.177)	decreased (0.018)
Zheng *et al.* (China) (29)	68	n.a.	decreased (< 0.05)	n.a	decreased (< 0.05)	n.a	decreased (< 0.05)	n.a.
n.a. – not available. NK – Natural killer cell. COVID-19 – coronavirus disease 2019.								

Along with the severity of COVID-19 disease, a progressive T cell exhaustion, mediated by the expression of some immune-inhibitory molecules, has been evidenced during the course of infection (*30*). It is well known that persistent stimulation by the virus may induce T cell exhaustion, leading to loss of cytokine production and cellular dysfunction (*31*). On the other hand, efficacious therapy with chloroquine was accompanied by an increased number of T cells and NK cells. Therefore, it might be hypothesized that in COVID-19 patients with severe pulmonary inflammation, SARS-CoV-2 correlates with functional exhaustion of cytotoxic lymphocytes at the early stage, which may result in disease (*29*). A low T cells count, an increase in naïve helper T cells, and a decrease in memory helper T cells were found in patients severely affected by COVID‐19 (*32*). Moreover, a lower level of regulatory T cells has been found in severe cases (*20*). Reconstitution of lymphocytes may be an important factor for recovery (*33*). Low lymphocyte count might be used by clinicians in risk stratification to predict severe and fatal COVID-19 in hospitalized patient.

### Neutrophils

Neutrophilia, except for patients with bacterial infections or superinfections, correlates with hyperinflammatory state and cytokine storm, an integral part of the pathogenic mechanism of COVID-19. Neutrophils are involved in many viral respiratory diseases associated with ARDS (*34*). A minority of patients present leucocytosis, supported by neutrophilia: this finding seems to correlate with a more severe course (*35*). As COVID-19 progresses, the number of circulating neutrophils gradually increases; thus, neutrophilia has been identified as a marker of severe respiratory disease and a poor outcome (*36*). Leukocytes and neutrophils were significantly higher in severe than in non-severe COVID-19 infected patients. Further, along with COVID-19 disease progression, both leukocyte and neutrophil counts increased in the severe groups (*37*).

In peripheral blood smear, morphological alterations in circulating neutrophils, such as the reduction of nuclear lobularity and the presence of heavy cytoplasmic granulations, have been described (*38*). These morphological changes were transient and reversible preceding the appearance of the large reactive atypical lymphocytes, characteristic of viral infections (*39*).

The increase of neutrophils has been reported not only in the bloodstream but also in the lung tissue (*40*). The increased infiltration of immature and/or dysfunctional neutrophil contributes to the abnormal lungs’ immune response in severe patients. Similar to other viral infections, SARS-CoV-2 infection promotes neutrophil extracellular traps release, which can contribute to tissue damage. Aberrant activation of neutrophils might exacerbate host response in COVID-19. Lung autopsies revealed the presence of neutrophils in lung capillaries and their extravasation into alveolar space (*41*). Neutrophils have a crucial role as drivers of hyperinflammation associated with COVID-19 disease via enhanced degranulation and cytokine production (*42*). On the day of hospital admission, the median neutrophil count in severe COVID-19 patients was found higher than in the moderate and mild groups. Further, an increase in neutrophil count from day 7 to day 9 after symptom onset was evidenced. Given the relationship between neutrophilia and poor outcomes, it has been proposed that the change in neutrophil counts in peripheral blood or tissues might be strictly associated with lung damage in COVID-19 patients (*43*). Confirmation of these findings might lead to targeting neutrophils and their recruitment mediators to reduce the severity of COVID-19.

### Neutrophil-lymphocyte and platelet-lymphocyte ratio

The neutrophil-lymphocyte ratio (NLR: absolute neutrophil count/absolute lymphocyte count) and platelet-lymphocyte ratio (PLR: absolute platelet count/absolute lymphocyte count) have been reported to be useful in the diagnosis, follow-up, and survey of various systemic inflammatory processes, such as cholangiocarcinoma, ischemic heart disease, acute pancreatitis, and as a prognostic marker of malignant tumours (*44*-*47*).

Neutrophil-lymphocyte ratio is elevated in the bloodstream of COVID-19 infected patients; Zhang *et al.* reported that NLR combined with IgG might be a better predictor than neutrophil count alone in predicting the severity of COVID-19 (*48*). Levels of NLR and PLR correlate with COVID-19 disease severity. Patients with severe disease had higher NLR and PLR values compared to non-severe diseases (*49*). Neutrophil-lymphocyte ratio and PLR have been considered independent factors associated with COVID-19 progression; however, the mechanisms behind this are not understood (*50*). At the early stage of COVID-19, the total number of leukocytes in peripheral blood is normal or decreases, while the lymphocyte count decreases (*51*). The initial and peak value of NLR in deceased patients were higher than in survivors (P < 0.001) (*52*). The increase of NLR means the progressive increase of neutrophils, and/or the decrease of lymphocytes. The increase of neutrophils often suggests an underlying bacterial infection, while the decrease of lymphocytes means a compromised system (*53*). Those suggest that it is necessary to pay attention to the COVID-19 patients with increased NLR (*52*). Worse outcomes were noted in patients presenting with a peak in the platelet count during the disease course, and the PLR at the time of platelet peak was identified as an independent prognostic factor for prolonged hospitalization (*54*). A case report describes NLR and PLR fluctuation during the progression of COVID-19 disease. From the admission, white blood cells, neutrophils, platelets, and the NLR gradually increased and reached a peak on the 14^th^ day, the PLR reached a peak on the 9^th^ day, while the number of lymphocytes did not reach the maximum value, but it showed only an upward trend. The NLR and the PLR gradually returned to normal after the patient’s improvement on the 14^th^ day (*55*). In COVID-19 patients, the levels of new serological biomarkers, including NLR and PLR, were elevated in the severe disease group compared to those in mild to moderate patients (*56*). In a different study, no statistically significant difference was observed between COVID-19 negative and positive patients, respectively, regarding lymphocyte and PLR values (P = 0.081), while a statistically significant difference (P < 0.001) was found between the test groups regarding NLR values (*57*).

In COVID-19 patients, increased ferritin and PLR were found to be independent predictors of the disease severity (*58*). SARS-CoV-2-triggered hyperinflammation seems to increase PLR, which promotes severe prognosis. Studies suggested that PLR was linked to inflammation and could predict mortality among haemodialysis patients (*59*). Neutrophil-lymphocyte ratio and PLR are easily obtained from a serum complete blood count with a differential profile. They serve as a function of relative neutrophilia, thrombocytosis, and lymphopenia. While many inflammatory markers like CRP, ESR, lactate dehydrogenase, ferritin, and PCT are frequently measured in COVID-19 patients, NLR and PLR can be easily calculated using the differential count and are cost-effective especially for many third world countries. Monitoring the predictors of severity may assist clinicians to identify and follow-up patients with a higher risk for progression.

### Monocytes

Monocytes constitute about 5-9% of the total peripheral leukocytes, remain in the circulation for 1-2 days, following which these cells may differentiate to tissue-resident macrophages (*60*). SARS-CoV2 infects CD14+ monocytes through angiotensin-converting enzyme (ACE2), but viral replication in these cells is usually low or undetectable (*61*, *62*). Anyway, the SARS-CoV-2-infected monocytes can produce large amounts of inflammatory mediators that support COVID-19-associated cytokine storm (*63*). Wen *et al.* revealed a predominant subset of CD14++IL1β+ monocytes in patients in the early recovery stage of COVID-19 (*64*). Zhang D *et al.* did not detect significant differences in the absolute number of monocytes between patients with COVID-19 and normal healthy individuals but identified an increased proportion of activated monocytes/macrophages in patients with COVID-19, and significant morphological and functional differences, which are more pronounced in patients requiring prolonged hospitalization and ICU admission. In particular, the numbers of circulating classical monocytes (CD14++CD16−) decrease, but the numbers of intermediate (CD14++CD16+) and non-classical (CD14+CD16++) monocytes increase in COVID-19 patients. Moreover, patients with COVID-19 have larger than normal monocytes, easily identified on forward scatter (FSC) analysis by routine flow cytometry, with the presence of a distinct population of monocytes with high FSC. These FSC-high monocytes are CD11b+, CD14+, CD16+, CD68+, CD80+, CD163+, CD206+ and secrete IL-6, IL-10, and TNF-alpha, consistent with an inflammatory phenotype. In contrast, patients with a high proportion of normal monocytes have a better prognosis with earlier recovery and discharge from the hospital. These findings appear to be relatively specific for COVID-19 as we have not seen a similar pattern in patients with other viral illnesses, such as H1N1 influenza and human immunodeficiency virus (HIV) (*61*). A significant expansion of populations of CD14+CD16+ monocytes producing IL-6 was observed in the peripheral blood of patients with COVID-19 in ICU compared with those patients who did not require ICU hospitalization. Moreover, monocytes from COVID-19 patients were found to be able to secrete granulocyte-macrophage colony-stimulating factor (GM-CSF) (*65*). As the monocyte is the pathogenic GM-CSF responsive cell that requires GM-CSF to promote tissue damage in both mouse and human, it has been suggested that the increasing number of GM-CSF+ monocytes and IL-6+ monocytes in the peripheral blood is responsible for inflammatory cytokine storms occurring in COVID-19 infection (*65*, *66*). SARS-CoV-2 infection of monocytes, which acts as antigen presenting cells, directly impairs the anti-viral adaptive immune responses. Thus, interfering with monocytes infection and subsequent activation of cytokine production and cytokine-mediated signalling pathways can also attenuate systemic hyperinflammation.

### Eosinophils

The role of eosinophils in the coronavirus-19 disease is unknown. Physiologically, tissue-resident eosinophils are mainly represented in the gastrointestinal tract and in the lung, where they have regulatory functions in protective immunity, and organ growth and metabolism (*67*). In a population of 140 patients infected with SARS-CoV-2 in Wuhan, 53% of the patients had eosinopenia at admission (*68*). Mu *et al.* evidenced a significant eosinopenia in 72/95 SARS-CoV-2 patients (P < 0.01). The absolute eosinophil count was 0.01 x10^9^/L and the eosinophil percentage was 0.3%. Moreover, eosinophil blood count progressively returned to normal together with clinical conditions improvement, while continued to decline for patients without clinical improvement (*69*). In a retrospective analysis by Tan *et al.*, decreased eosinophils were detected in 33 out of 40 patients with COVID‐19 on admission, while eosinophils were inversely related to the severity of the disease, according to the Spearman’s correlation coefficient (*r*_s_ = -0.462; P = 0 .003). Furthermore, the patients’ peripheral blood eosinophils returned to normal values before the discharge, suggesting a putative role of eosinophils in predicting the prognosis of the patient (*70*).

Eosinopenia as a response to infection was first described in Zappert (*71*). The pathophysiology for eosinopenia in COVID-19 remains unclear but is likely multifactorial and related to the migration of eosinophils to the inflammatory site, inhibition of eosinophil mobilization from the bone marrow, blockade of eosinophilopoiesis, reduced expression of chemokine receptors/adhesion molecules, and/or direct eosinophil apoptosis induced by IFN-I released during the acute phase of inflammation (*72*, *73*).

### Basophils

*In-vivo*, basophils leave the circulation and migrate to inflammatory sites during allergic inflammation and infection, enhancing immunological memory responses by binding antigens on their surface (*74*). Secretion of IgM or a class switch to IgG or IgA by B cells is enhanced by activated basophils (*75*).

Rodriguez *et al.* demonstrated that basophils are depleted during acute and severe COVID-19, thus suggesting that the degree of basophil depletion may influence the efficacy of IgG responses to SARS-CoV-2 (*76*). Lower basophils were found in COVID-19 and influenza groups compared to healthy controls (*77*). Li *et al.* found that in the early stages of the disease, basophils counts were lower in COVID-19 patients than in controls (*78*). It is known that both basophils and eosinophils can produce IL-4, which is an important cytokine to stimulate the proliferation of activated B and T cells (*79*). Therefore, the decrease of basophil and eosinophil counts in COVID-19 patients may further explain the decrease of lymphocyte count. In a cohort of 452 patients affected by severe COVID-19 disease, higher leukocyte, and neutrophil counts and a lower percentage of basophils were found (*20*). Interestingly, low basophil counts were associated with viral infections in immunocompromised patients (*80*). On the other hand, it has been demonstrated that several HIV proteins can interact with different surface receptors on human basophils; also, HIV-infected basophils were found in the peripheral blood of acquired immunodeficiency syndrome patients (*81*, *82*). Based on these considerations, a mechanism of viral encapsulation in basophils might be postulated as one possible explanation of low basophil count in patients affected by COVID-19 infection.

### Red blood cells and haemoglobin

A significant impact of SARS-Co-V2-infection on red blood cell (RBC) structural membrane homeostasis at the protein and lipid levels was demonstrated. RBCs from COVID-19 patients had increased levels of glycolytic intermediates, accompanied by oxidation and fragmentation of membrane proteins. Thus, COVID-19 impacts two critical mechanisms that finely tune red cell membranes and haemoglobin oxygen affinity. RBCs from COVID-19 patients may be incapable of responding to environmental variations in haemoglobin oxygen saturation when traveling from the lungs to the bloodstream and, as such, may have a compromised capacity to transport and deliver oxygen (*83*). COVID-19 patients show higher levels of nitric oxide (NO) inside RBC compared to non-COVID-19 hypoxemic patients, but the mechanism(s) causing the accumulation of intracellular NO in RBC of COVID-19 patients is still unclear (*84*). Autoimmune haemolytic anaemia (AIHA) was recently described in COVID-19 patients (*85*). Autoimmune haemolytic anaemia causes platelet cell death and RBCs can also modulate platelet activity through either chemical signalling or direct RBC-platelet interactions. Thus, evidence for haemolysis may account for the microvascular coagulation seen in COVID-19 patients. Berzuini *et al.* reported the observation that about half of patients with COVID-19 tested at their blood center had a positive direct antiglobulin test (DAT). However, eluates did not react with any test cells but did react with red cells from patients with COVID-19 that were DAT negative. This suggests that COVID-19 may modulate the red cell membrane and present novel antigenic epitopes (*86*).

Patients with COVID-19 present decreased haemoglobin concentration and pathologically increased concentrations of ferritin. Wang *et al.* reported reduced haemoglobin concentration (< 110 g/L) in 19% of the hospitalized patients (*87*); while Huang *et al.* reported reduction in haemoglobin concentrations in 38% of the study population admitted to the hospital (*88*). In this case, the higher incidence of a low haemoglobin concentration in COVID-19 patients described by Huang might be explained by the difference in age structure between the populations from the two studies. In fact, Huang *et al.* reported data from a population aged 56 (26-88), while the study population described by Wang *et al.* had a median age of 42 (33–53). Haemoglobin concentration below 110 g/L was associated with disease progression (*89*). On the other hand, Cai *et al.* discovered no associations between haemoglobin concentration and risk of admission at ICU (*90*). Taneri *et al.* analysed data from 189 studies and 57,563 COVID-19 patients across all ages, founding a pooled mean haemoglobin concentration of 129.7 g/L, which decreased with older age and a higher proportion of comorbid illness and disease severity. They also found increased values of ferritin in most patients, mainly in males, the elderly, and individuals with hypertension (*91*). Based on data from 54 observational studies, including 24,262 COVID-19 patients, pooled mean ferritin concentration in COVID-19 patients was 777.33 ng/mL Moreover, the mean difference in serum ferritin was higher in severe COVID-19 individuals compared to moderate cases. A linear trend with ferritin concentrations between severe and moderate COVID-19 cases increasing with advancing age was observed, while non-linear trends were observed between patients’ characteristics and ferritin concentration among survivors and non-survivors. Odds in hospital death were higher among patients with ferritin above 300 ng/mL compared to those with serum ferritin ≤ 399 ng/mL (odds ratio 9.10 [(95% CI 2.04 to 40.58, P = 0.004]). Concentrations of serum ferritin were elevated in non-survivors compared with survivors (562 ng/mL *vs* 492 ng/mL) throughout the clinical course and were increased with disease deterioration (*92*). In another study, ferritin was significantly higher in severe cases (*93*). Both anaemia and hyperferritinemia, regardless of the underlying pathology, are associated with increased mortality (*94*). Haemoglobin concentrations were found lower with older age, a higher percentage of subjects with diabetes, hypertension, and overall comorbidities, and admitted to intensive care. Ferritin concentration increased with older age, increasing proportion of hypertensive study participants, and increasing proportion of mortality. Compared to moderate cases, severe COVID-19 cases had lower haemoglobin and RBC and higher ferritin and red cell distribution width (RDW) (*95*).

Anaemia could be the result of a sideroblastic-like anaemia pattern arising from alterations in iron metabolism, while increased ferritin could be indicative of a strong inflammatory reaction in COVID-19 or related to viral entry into the human body and its impact on iron metabolism. Functional iron deficiency was associated with significantly poorer clinical conditions and a longer hospital stay but was not linked to an increased risk of in-hospital death, ICU admission, or the need for mechanical ventilation. No significant relations were found between mortality rate and elevated ferritin concentrations. However, elevated ferritin concentrations were associated with longer hospital stays and increased risk for ICU admission and the need for mechanical ventilation (*96*). In COVID-19 infection, inflammation is related to iron metabolism biomarkers, and haemoglobin. Anaemic patients had significantly higher CRP, IL-6 and PCT levels. Accordingly, haemoglobin concentrations were only significantly correlated with CRP and tended to be related to IL-6, and PCT concentrations. In addition, patients with functional iron deficiency had significantly higher CRP, IL-6, and PCT concentrations when compared to patients with absolute iron deficiency or no iron deficiency. Accordingly, ferritin positively correlated with CRP, IL-6, and PCT concentrations. Taken together, these data are in accordance with the association of altered iron homeostasis with more advanced inflammation, the latter promoting lung injury and respiratory failure (*97*). Nonetheless, both anaemia and inflammation-induced alterations of iron homeostasis are important clinical predictors for risk stratification of SARS-CoV-2-infected patients.

Severe acute respiratory syndrome coronavirus 2 was shown to interfere with haemoglobin at erythrocyte and bone marrow level; the new SARS-CoV2 attack to bone marrow erythroblasts (*98*). Moreover, studies evidence an increased RDW in patients with COVID-19, thus suggesting the existence of underlying erythroid myelodysplasia, as RDW is higher when immature cells are produced (*99*). It has been found that SARS-CoV-2 interacts through ACE2, CD147, CD26, and other receptors located on erythrocytes and/or blood cell precursors, thus causing viral endocytosis. Consequently, the virus would attack the heme on the 1-beta chain of haemoglobin, inducing haemolysis and forming a complex with the released heme, producing a quote of dysfunctional haemoglobin (*100*).

## Limitations of the study

A large proportion of the primary research is based on Asian patients; therefore, further verification is needed in populations in other areas. Eventually, further investigations are needed to assess geographic variability among blood parameters. This is a review of the current scientific literature with no statistical outcome measures. Therefore, these results may not be generalizable to all populations. Finally, the rapidly evolving nature of COVID-19 and the continuous discovery of novel data are additional limitations of this article.

## Conclusions

Coronavirus disease 2019 has prominent manifestations from the hematopoietic system. Common haematological abnormalities have been identified in COVID-19 patients. Since the early stage of the disease, not only the platelets and lymphocytes but also haemoglobin, eosinophils, and basophils present a marked decrease, associating with the disease severity and clinical outcome. At the moment, the kinetics of monocytes in COVID-19 infection is still undefined, as SARS-CoV-2 infection of monocytes seems to directly impair the anti-viral adaptive immune responses. Finally, an increase of neutrophils and the two markers NLR and PLR seem to correlate with progressive disease. Careful evaluation of laboratory indices at baseline and during the disease course can assist clinicians in formulating a tailored treatment approach and promptly provide intensive care to those who are in greater need.

This review has emphasized the importance of laboratory information in the management of COVID-19, further studies are worth describing the association between the dynamic haematological responses and the progression and outcome of the disease.
